# Attitudes of Australian Veterinary Professionals to Diagnosing and Managing Canine Cognitive Dysfunction

**DOI:** 10.3390/vetsci12030272

**Published:** 2025-03-13

**Authors:** Auréa Brisset, Tracey L. Taylor, Tiphaine Blanchard, Eduardo J. Fernandez, Susan J. Hazel

**Affiliations:** 1 Ecole Nationale Vétérinaire de Toulouse, 31300 Toulouse, France; aurea.brisset@gmail.com (A.B.);; 2 School of Animal and Veterinary Science, Roseworthy Campus, The University of Adelaide, Adelaide, SA 5005, Australia; tracey.taylor@adelaide.edu.au (T.L.T.); eduardo.fernandez@adelaide.edu.au (E.J.F.); 3 GenPhySE, Université de Toulouse, French National Research Institute for Agriculture, Food and Environment (INRAE,) 31326 Castanet Tolosan, France

**Keywords:** canine cognitive dysfunction, canine, veterinarian, burden of care

## Abstract

Canine cognitive dysfunction (CCD) is a neurodegenerative disease likely to affect 14% to 35% of dogs over the age of eight years. Although it can be associated with a high burden of care in owners of affected dogs, there is a lack of knowledge of how veterinarians diagnose and manage the disease. The aim of this study was to determine how veterinary professionals (veterinarians and veterinary nurses/technicians) manage CCD and their attitudes towards the disease using an anonymous online survey. Chi-square tests were used to assess differences according to years of experience, and multiple correspondence analysis was used to analyze clusters according to age, gender, and experience. One hundred and four responses were obtained: 73 veterinarians and 31 veterinary nurses/technicians. Veterinarians diagnosed CCD based on their own experience or by excluding other diseases (27/73, 37% and 25/73, 34%, respectively) and mostly diagnosed a few cases/year (34/73, 47%) or a few in their career (19/73, 26%). CCD was managed using specific medication or environmental changes (57/73, 78% and 58/73, 79%, respectively). Over half of veterinarians agreed/strongly agreed that they were confident in diagnosing CCD (49/73, 67%) and in giving advice on symptom management (53/73, 71%), but only 14/31 (45%) of veterinary nurses/technicians agreed that veterinarians in their practice actively treated dogs with CCD. Participants mostly agreed that CCD is associated with a large burden of care for owners, but less than half measured this burden of care. Although veterinary professionals are aware of CCD, the low rate of diagnosis suggests many dogs are undiagnosed. Increased awareness of the disease by the veterinary profession will enhance human and dog welfare.

## Simple Summary

Canine cognitive dysfunction (CCD) is a neurodegenerative disease similar to Alzheimer’s disease (AD) likely to affect 14% to 35% of dogs over the age of eight years. Clinical signs are highly varied and include inactivity, inattention, anxiety, incontinence, wandering, and sleep–wake cycle disorder. The aim of this study was to determine how veterinary professionals (veterinarians and veterinary nurses/technicians) manage CCD and their attitudes towards the disease using an anonymous online survey. Veterinarians diagnosed CCD based on their own experience or by excluding other diseases and mostly diagnosed a few cases per year or a few in their career. CCD was managed using specific medication, diet adjustments, or environmental changes. Although veterinary professionals are aware of CCD, the low rate of diagnosis suggests many dogs are undiagnosed. Increased awareness of the disease by the veterinary profession will enhance human and dog welfare.

## 1. Introduction

Canine cognitive dysfunction (CCD) is a neurodegenerative disease in older dogs similar to Alzheimer’s disease (AD) in humans [1,2]. The prevalence of CCD is estimated between 14% and 35% in dogs over the age of eight years [3,4,5,6]. Age is the main risk factor for CCD; with modern veterinary medicine extending the average lifespan of dogs [5,7,8], it is likely that the number of dogs with CCD will increase. However, CCD may be wrongly confused with normal aging by owners and veterinarians and is likely to be underdiagnosed [6]. Clinical signs are highly varied [7] and include inactivity, inattention, anxiety, incontinence, wandering, and sleep–wake cycle disorders [4]. While these signs may also be seen in normally aging dogs, they are more severe in CCD. The disease is still not well understood and deserves more attention from the veterinary community and dog owners, who play a key role in the management and prevention of the disease.

The pathophysiology of CCD is multifactorial, and there is no consensus on diagnosis or treatment. Diagnosis is mainly based on information provided by the owner, in particular, the report of clinical signs [4]. The history and clinical examination, combined with the exclusion of other conditions likely to mimic the clinical signs of CCD, can lead to a strong suspicion of CCD. A number of assessment questionnaires are available to help veterinarians and owners screen for the disease, including CCDR [9], CADES [4], and DISHAA [10], which can be helpful for diagnosis and monitoring changes over time.

Some behavior tests can also be performed to assess cognitive status. However, CCD is suspected to be largely underdiagnosed [6]. Treatment is palliative and aims to improve the quality of life by alleviating clinical signs and maintaining cognition, but it is not curative and requires a holistic approach to the owner–pet dyad. Several specific treatments have been validated for the treatment of CCD, and many non-specific drugs are described to have an effect on clinical signs [4,11]. A wide range of non-medical treatments have also proven effective: dietary adjustments, food supplements, environmental modifications, exercise, and alternative medicine [12]. Combinations of these treatments are likely to provide the best results.

Behavior changes due to CCD may alter the relationship between owner and pet by increasing an owner’s burden of care. Caregiver burden is a complex, multidimensional concept defined as an individual’s response to challenges they face when providing care for a sick family member [13]. It is well described for caregivers of people with dementia [14,15], and a clinical level of burden is reported in 50% owners of pets with terminal diseases [16] and 16% of caregivers of dogs suffering from CCD [17]. One of its implications is reduced adherence to the treatment plan and, hence, deterioration in disease management. Burden may also ultimately lead to early euthanasia, which is regrettable because this disease has no impact on the longevity; dogs that are well cared for can continue to live long and high-quality lives [7].

The aim of this study is firstly to assess how veterinarians manage CCD: how they diagnose the disease, how often, and how they treat it to explore the possible underdiagnosis of the disease or other limitations of its management. Secondly, our aim is to assess the perception of the disease by veterinarians and veterinary nurses and technicians and their attitude towards the owners caring for a senior dog with the disease. This assessment also aims to provide insights into factors that may influence the effective management of CCD.

## 2. Materials and Methods

### 2.1. Survey

The first part of the questionnaire addressed demographics. Demographic questions included gender (female, male, non-binary, and prefer not to say), age (18–24, 25–34, 35–44, 45–54, 55–64, and 65+), and if they were a veterinarian or a veterinary nurse or technician. The following questions for the veterinarians included year of graduation (1957–1968, 1969–1979, 1980–1990, 1991–2001, 2002–2012, and 2013–2023), country of graduation (Australia/New Zealand or overseas), type of veterinary practice (small animal or mixed), and postgraduate specialization (no, membership, fellowship, internship, residency, or other). For the veterinarians, an additional question asked if they had ever lived with a dog with dementia (yes/no). For the veterinary nurses/technicians, there was a single question on how long they had worked as a veterinary nurse or technician (less than 12 months, 1 year to less than 5 years, 5 years to less than 10 years, and 10 years or more). The next group of questions asked about how CCD was managed in the veterinary clinic: frequency of diagnosis, how they diagnose, treatments used, and programs provided by the practice for older dogs (Table 1).

The next set of questions asked about attitudes to dogs with dementia and their owners by assessing the level of agreement to statements on a five-level Likert scale: from “strongly disagree” to “strongly agree” (Table 2).

The final set of questions asked about attitudes to owners of dogs with dementia. Questions were on the same five-level scale from “strongly disagree” to “strongly agree” (Table 3).

### 2.2. Recruitment

The survey recruited veterinarians, veterinarian nurses, and technicians, 18 years of age or older, and currently practicing in Australia. Only complete responses to the questionnaire were included in the study. The survey was made available online via the software REDCap 14.5.28 (Research Electronic Data Capture) from 1 May 2024 to 24 June 2024. The questionnaire was circulated via social media (Facebook™), e-mail communication to veterinary clinics, and through passive snowballing. The study was approved by the Human Research Ethics at the University of Adelaide (H-2024-061).

### 2.3. Statistical Methods

Data were directly imported from RedCap to Microsoft Excel. Only complete responses were included in the analysis, with incomplete responses removed. Free-text questions were grouped thematically. Quantitative Likert-type questions were grouped into three categories: negative agreement (“disagree” and “strongly disagree”), neutral agreement (“neither agree nor disagree”), and positive agreement (“agree” and “strongly agree”). The primary focus of our research was whether respondents agreed or disagreed with the statements, rather than distinguishing subtle differences between “agree” and “strongly agree” or “disagree” and “strongly disagree.”

Chi-square tests were performed to explore veterinarian’s management of CCD in dogs and their attitude to the disease according to their professional experience as well as their experience of having lived with a dog with CCD. We hypothesized that longer professional experience of veterinarians is associated with greater confidence in treating dogs with CCD, being more proactive in diagnosing the disease, being more likely to use validated diagnostic tools, and being more active in treating with specific medication and that less-experienced veterinarians would be more likely to prefer treating other diseases rather than CCD.

Statistical significance was set at *p* < 0.05. Where multiple comparisons were made, a Bonferonni adjustment was made.

Analysis of clusters was performed to determine if different groups of veterinarians vary in their knowledge, diagnosis, and management of CCD. Statistical analyses were performed using R software version 4.2.2 [18] with the FactoMineR package version 2.11 [19]. A multiple correspondence analysis (MCA) was conducted with characteristics of the veterinarian added as supplemental variables (e.g., age and gender) and variables related to their knowledge, diagnosis, and management included as active variables. MCA is an exploratory technique used to project variable categories into a low-dimensional space defined by synthetic axes (dimensions), where associations between variable categories and their frequencies determine their proximity on the plot. Based on the MCA results, veterinarians with similar CCD knowledge, diagnosis, and management approaches were grouped into clusters using Hierarchical Cluster Analysis (HCA) with Ward’s method. The ideal number of clusters was chosen by visualization of the inertia gain. To identify the variables that best characterized each cluster, a hypergeometric test was performed to assess the over-representation of specific variables within each cluster [20]. A characteristic was considered associated to a cluster when the v-test value was >2.0.

## 3. Results

### 3.1. Demographics

One hundred and four participants responded to the survey. Among them, 73 were veterinarians (70%; V), and 31 were veterinary nurses or technicians (30%; VN/T). Most veterinarians were female (84%, n = 61), graduated in Australia or New Zealand (92%, n = 67), and worked in a small animal practice (84%, n = 61). Thirty-seven percent (n = 27) of V participants had lived with a dog with CCD. The majority of VN/T participants were also female (97%, n = 30) (Table 4).

### 3.2. Management of CCD

CCD was mainly diagnosed by V using their own experience (37%, n = 27) or by the exclusion of any other disease (34%, n = 25) (Table 5). Three participants did not diagnose CCD at all (4%, n = 3). The frequency of the diagnosis of CCD was a few times per year in 47% (n = 34) of V participants, 26% (n = 19) diagnosed only a few in their career, and 26% (n = 19) a few per month. Only one participant diagnosed CCD a few times a week. The management of CCD was mainly performed by V with specific medication for CCD (78%, n = 57), environment changes (79%, n = 58), anti-anxiety treatments (73%, n = 53), and food supplements (66%, n = 48). Programs provided by the veterinary practices for older dogs included general health (56%, n = 41) and obesity (30%, n = 22), but almost half of V reported their practice provided no programs for older dogs (45%, n = 33).

A majority (68%, n = 21) of VN/T participants were involved in the management of CCD in dogs. In their practice, CCD was mostly managed by making environmental changes (77%, n = 24), less frequently using physiotherapy (29%, n = 9), or referral to a dog trainer (19%, n = 6). Sixteen percent (n = 5) reported nothing was used in their practice for the management of dogs with dementia. Among those who used other types of management (n = 12), medical treatment was mentioned by 67% (n = 8), as well as dietary supplements (50%, n = 6). One VN/T reported their practice used cognitive enrichment as a treatment for CCD. According to the VN/T, the veterinary practices they worked in provided general health checks for older dogs (81%, n = 25) and obesity checks (55%, n = 17) with 19% (n = 6) reporting that their practice does not provide any programs for older dogs. Massages were provided for older dogs in one VN/T practice.

### 3.3. Attitude Towards the Disease

**Figure 1:**
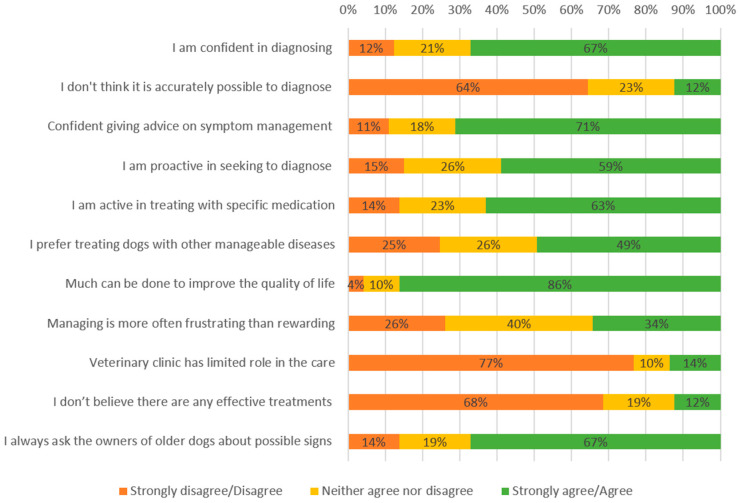
Veterinarian confidence in diagnosing and treating canine cognitive dysfunction (CCD).

Over half of V agreed/strongly agreed that they were confident in diagnosing dementia in dogs (67%, n = 49) and were confident in giving advice on symptom management (71%, n = 52) (Figure 1). Almost two-thirds believed the disease could be accurately diagnosed (64%, n = 47), most agreed that much can be done to improve the quality of life of CCD patients (86%, n = 63), that veterinary clinics have a role in the care (77%, n = 56), that there are effective treatments (68%, n = 50), and that they were active in treating dementia with specific medication (63%, n = 46). Although a majority of V participants reported that they always asked owners about possible signs of the disease (67%, n = 49), fewer considered they were proactive in seeking to diagnose the disease (59%, n = 43).

**Figure 2:**
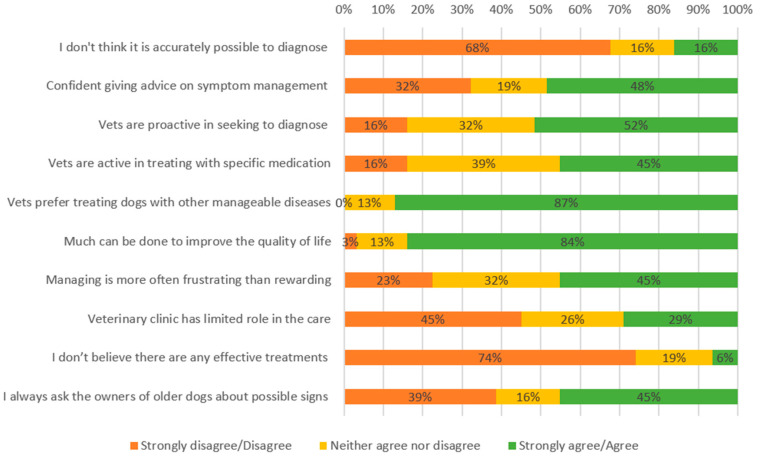
Veterinary nurse/technician confidence in diagnosing and treating canine cognitive dysfunction (CCD).

VN/T participants were less confident than V participants in giving advice on symptoms management, as only 48% (n = 15) reported being confident (versus 71% of V). VN/T mostly disagreed (68%, n = 21) that diagnosis cannot be accurately made, believed much can be done to improve the quality of life (84%, n = 26), and mostly believed that there are effective treatments (74%, n = 23) (Figure 2). However, only 45% (n = 14) of VN/T think veterinarians in their practice actively treat dogs with CCD with specific medication and 52% (n = 16) that they were proactive in seeking to diagnose the disease. Finally, most VN/T participants considered veterinarians in their practice preferred treating other manageable diseases than CCD (87%, n = 27).

### 3.4. Attitude Towards Owners of Dogs with CCD

**Figure 3:**
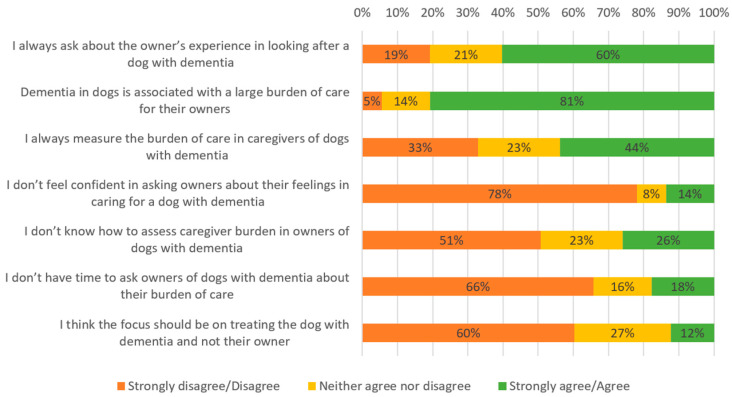
Attitude to interactions with the owners of dogs with CCD—V.

**Figure 4:**
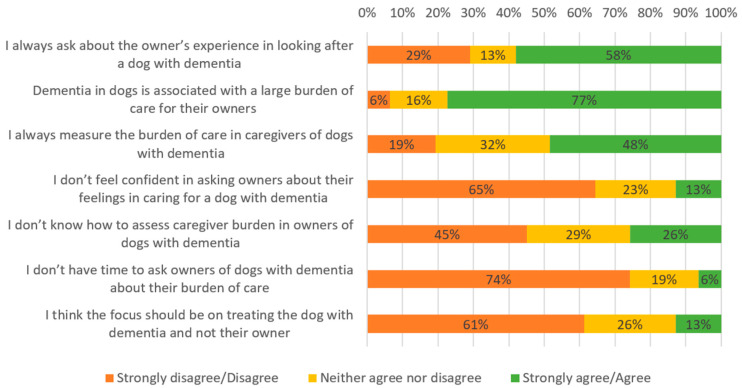
Attitude to interactions with the owners of dogs with CCD—VN/T.

Responses relating to attitudes to interacting with the owners of dogs with CCD were similar between V and VN/T (Figure 3 and Figure 4). Participants mostly agreed/strongly agreed that dementia was associated with a large burden of care (V: 81%, n = 59; VN/T: 77%, n = 24) and always asked about the owner’s experience in looking after a dog with dementia (V: 60%, n = 44; VN/T: 58%, n = 18), but fewer agreed/strongly agreed that they measured the burden of care of owners (V: 44%, n = 32; VN/T: 48%, n = 15). Few participants reported a lack of confidence (V: 14%, n = 10; VN/T:13%, n = 4), knowledge (V: 26%, n = 19; VN/T: 26%, n = 8), or time (V: 18%, n = 13; VN/T: 6%, n = 2) when asking owners about their burden of care. Finally, most participants agreed that the management of the disease should focus on the patient as well as their owner (V: 60%, n = 44; VN/T: 61%, n = 19).

### 3.5. Factors Influencing Management and Attitude

The Chi-square tests showed no significant difference in the management and attitudes of veterinarians according to their professional experience or experience of living with a dog with CCD (FDRadjusted *p* > 0.005). However, more experienced practitioners tended to be less likely to prefer treating any other diseases (20%) than less-experienced ones (57%) (*p* = 0.011) and to treat with specific medication (87% versus 57% for those who graduated after 2002, *p* = 0.033).

The MCA was conducted on the 27 first factorial axes, accounting for 80.5% of the variance. One veterinarian was identified as a satellite and excluded from the analysis. The HCA analysis resulted in the identification of two clusters (or profiles) of veterinarians based on their knowledge, diagnosis, and management of CCD.

Characteristics associated with each cluster are presented in Table 6.

**Figure 5:**
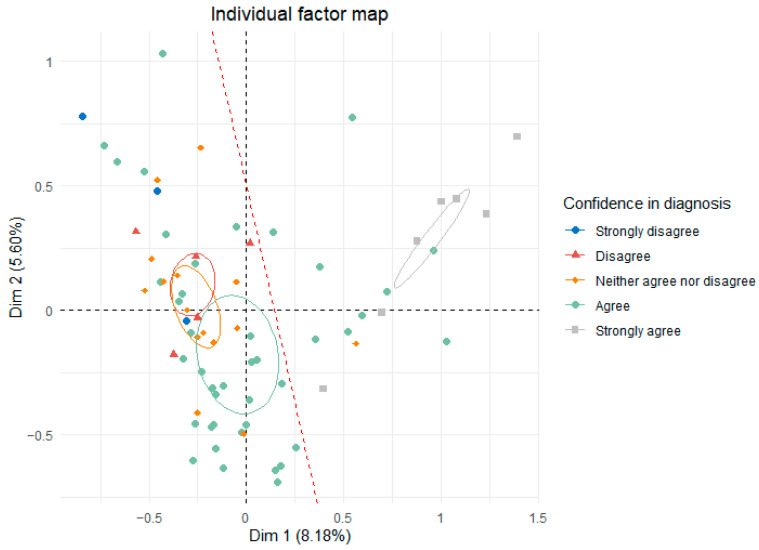
Individual factor map of the MCA on veterinarians’ responses regarding their confidence in diagnosing the disease, with the red line distinguishing the Junior group (left) from the Senior group (right). The ellipses represent confidence intervals for the Likert-type responses.

**Figure 6:**
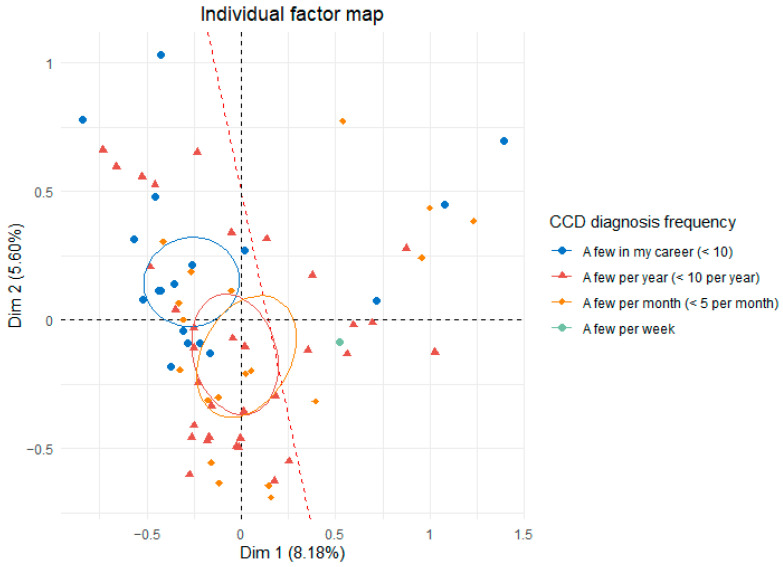
Individual factor map of the MCA on veterinarians’ responses regarding their frequency of diagnosis, with the red line distinguishing the Junior group (left) from the Senior group (right). The ellipses represent confidence intervals for the Likert-type responses.

Cluster 1 (Junior group) was younger and had graduated more recently than Cluster 2 (Senior group) and was less confident in diagnosing and managing the disease and, therefore, also less proactive in diagnosing it (Figure 5). However, no group diagnosed CCD more frequently (Figure 6). Regarding treatment, all participants who did not treat CCD were part of the Junior group, regardless of the type of treatment (medication, environmental changes, physiotherapy, or diet changes).

Twenty (19%) participants provided comments in the final free text box. Some expressed concerns on difficulties relating to CCD in dogs:

*“More treatment modali* *ties are needed for CCD”;*

*“I feel this is a neglected area and could be managed be* *tter”;*

*“It would be wonderful if we had more ways to prevent CCD”.*

Participants also expressed feelings about the owners being obstacles to good management:

*“Clients generally deny, dismiss or decline treatment as it’s seen as ‘op* *tional’”;*

*“I find it is the owners that don’t want to spend a lot of money on an elderly dog and don’t want to put the time in”.*

## 4. Discussion

The aims of this study were to assess how veterinary professionals manage dogs with CCD. This included how veterinarians manage and diagnose CCD, the perception of the disease by veterinarians and veterinary nurses/technicians, and attitudes towards the owners caring for a senior dog with the disease.

### 4.1. Management of CCD

#### 4.1.1. Diagnosis

A main finding of this study is that veterinarians were diagnosing CCD in dogs infrequently, with 73% (n = 53) of respondents diagnosing CCD only a few times a year or less. However, based on the following calculations, veterinarians should see a dog with dementia every four to ten days that they are consulting. This estimate is based on veterinarians in Australia performing 13 consultations per day [21]. We could assume that half of these consults are for dogs based on pet ownership data, giving six dogs per day. According to the 2023 Victorian pet census, 47% of the state’s dog population is over 5 years old, and 18% is over 10 years old [22]. If we consider that all age groups are equally represented in consultations—though older dogs may require more care and, therefore, be more represented—veterinarians should be seeing one dog over 10 years of age per day. Finally, given that the prevalence of CCD in dogs aged eight years or over is between 14% and 35% [3,4,5,6], this means that the average veterinarian should see a dog with dementia every four to ten days that they are consulting. This contrasts with 73% of veterinarians in the current survey diagnosing CCD in dogs a few times per year or less and confirms an earlier study that suggested the prevalence of CCD in a large survey of community-based dogs to be 14.2%, although only 1.9% were diagnosed with CCD by a veterinarian [6].

The low diagnosis of CCD in dogs contrasts with the 67% (n = 49) of veterinarians who considered that they always asked for clinical signs and 59% (n = 43) who believed that they were proactive in looking for the disease. In addition, although participants in the Senior group tended to be more proactive and confident in diagnosing the disease, they did not diagnose CCD more often than the Junior group. This mismatch suggests there may be a lack of knowledge about the prevalence of the disease by veterinarians. Wallis et al. (2023) in a qualitative investigation on the veterinary professional experience of and attitude to aging in dogs described that veterinarians did not consider CCD as a significant issue in older dogs [8]. The apparent underdiagnosis combined with veterinarians not considering CCD as a significant issue in older dogs may result in reduced dog welfare and human wellbeing. If the signs of CCD are mistaken for normal aging, owners may not be offered treatments such as CCD specific or anti-anxiety medications, which could improve the welfare of the dog. The lack of treatment may also mean owners are left with a dog pacing and not sleeping at night, negatively affecting their wellbeing.

Another major finding of our study was that specific diagnostic tools such as validated scales are underutilized by the practitioners surveyed, and most of them only use their own experience (37%, n = 27) or only make a diagnosis by excluding any other illness (34%, n = 25). Although the exclusion of any other disease is important, diagnosis would be more effective, more precise, and earlier if specific tools such as rating scales were used. The training of veterinary students or veterinarians in practice in the use of validated scales might enhance outcomes for dogs and their owners. This lack of diagnostic testing is not the only obstacle that can lead to the underdiagnosis of CCD.

#### 4.1.2. Why Is CCD Underdiagnosed?

As with any other disease, communication between the veterinarian and the owner is critical. However, unlike any other disease, the diagnosis of CCD is primarily based on the report of clinical signs from the owner [4,11]. The reduced reporting of clinical signs can be a major obstacle to diagnosis. This can be seen with arthritis in dogs, as few owners attributed early behavioral changes to osteoarthritis, meaning some waited months before seeing a veterinarian. A combination of short consultation lengths, poor awareness by the veterinarian of the knowledge levels of the owner, and a lack of recognition of the importance of the owner’s prior knowledge and beliefs all contribute to consultations challenging to both veterinarian and owner [23]. It is also the case in pain management, where subtle behavioral changes may be easily overlooked by owners and members of the veterinary community [24]. The lack of communication between owners and veterinary staff is a well-described hindrance to good care, especially in the case of geriatric populations [23,25]. Communication between veterinarians and owners has been described as the most important factor for promoting senior dog health [8]. A future focus on educating veterinarians and owners on the high prevalence of CCD in older dogs and encouraging veterinary staff to routinely use validated scales, such as CADES, in older dogs, will help to increase the low rates of CCD diagnosis.

**Figure 7:**
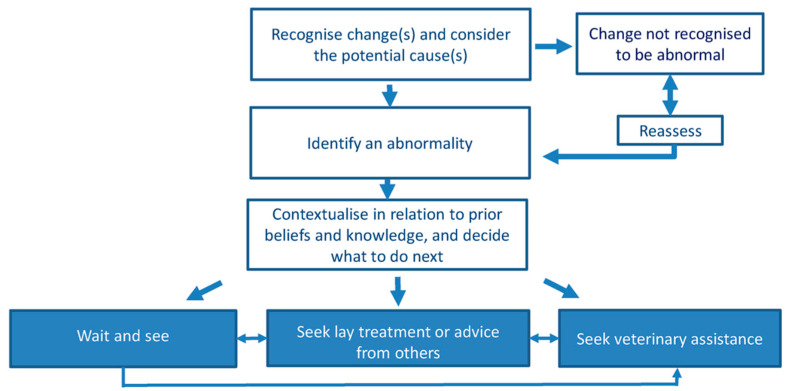
Schematic diagram summarizing the key decision-making steps described by owners when deciding what to do with a dog that might have osteoarthritis [23].

Furthermore, CCD can often be confounded with normal aging, even by members of the veterinary community, due to a lack of education on the subject [4,25]. Indeed, an owner’s decision-making can be largely influenced by their perception of the disease, including their beliefs and previous experiences [23,25]. Some owners may not recognize clinical signs as abnormal and consider it is normal aging, while others may recognize them but not report them because they are concerned about costs [26] or the outcomes of the consultation, for instance, the suggestion of euthanasia [27]. A “folks model of illness” has been described in humans and is the theory that there are three outcomes for health decisions: (1) “wait and see”, (2) “lay treatment including asking others for advice” and (3) “seek help from a medical professional” [28] (Figure 7). Evidence of such an interaction between beliefs and outcomes has also been reported for animal diseases, such as osteoarthritis in dogs and colic in horses [23]. However, effective communication strategies on the part of the veterinarian can compensate for the lack of reporting of clinical signs and enable earlier identification of disease. Therefore, improving veterinarians’ communication skills and raising awareness of the disease among clients and members of the veterinary community may be key to improving the diagnosis and care of the disease [8]. Taking osteoarthritis as an example, awareness campaigns aimed at dog owners have been launched and could also be useful in the case of CCD [23]. Regarding the care of aged dogs in general, it has been suggested that giving owners standardized tools for assessing a senior dog, with indications on the important signs to look for, would be beneficial and could guide discussion in consultations [8].

Veterinarians may also lack knowledge of CCD in dogs and, therefore, not be proactive in actively seeking it out during consultations. Our Hierarchical Cluster Analysis shows that although there was no difference in how many cases of CCD were diagnosed per year in the younger and older groups, the Junior group were less likely to treat dogs with CCD and also less confident in diagnosing and managing the disease. In the area of veterinary nutrition, a main barrier to nutrition communication is veterinarians’ confidence in knowledge of nutrition [29]. Keeping up to date with current research is essential for veterinarians but lack of time is described as an obstacle and may lead to gaps in knowledge [8]. Our findings suggest training should be particularly targeted at more recent graduates. Fortunately, studies have shown that training can have a positive effect on knowledge and confidence in the field of dementia in humans [30]. We can assume that further education and postgraduate training would increase veterinarians’ confidence in dealing with CCD as well, making them more proactive in managing the disease.

#### 4.1.3. Treatment

The main treatments used by the veterinarians surveyed were specific medications for CCD: selegiline, propentofylline (78%, n = 57), environment changes (79%, n = 58), anti-anxiety treatments (73%, n = 53), and food supplements (66%, n = 48), which are the main treatments indicated in the literature [4,12,31,32]. The use of medical treatments is well described in the literature, whether specific, such as selegiline or propentofylline, or non-specific, such as anti-anxiety medication, as well as GABAergic drugs (e.g., gabapentin), which were also mentioned by participants [4]. Although being validated for CCD, selegiline has variable results between patients [4]. Additionally, though short-term improvement of cognition has been observed both in dogs and humans, previous studies in humans with AD showed there were indeed short-term benefits but no clinically significant long-term effects [33,34]. Its use also raises concern as it cannot be used simultaneously with anti-anxiety medication, including selective serotonin reuptake inhibitors (fluoxetine) and drugs that might enhance serotonin transmission (buspirone and trazodone) [35]. Veterinarians may then have to choose between anti-anxiety medication or specific medication to CCD, with the limitations of efficacy it has.

Food supplements are also broadly described in the literature to be efficient, especially for antioxidants [4,36,37,38,39] and medium-chained triglycerides [4,32,35,40,41], but also N-acetyl-cysteine and phosphatidylserine [4,12,41]. Supplementation products specific for CCD are available on the market as well as kibbles, which are specially developed and could help delay the loss of cognition [39]. Environmental changes and enrichment are also major tools for the management of CCD, with several studies showing that they improve neuronal plasticity, reduce neuronal loss in the hippocampus, and therefore preserve cognition, while reducing cognitive impairment and delaying and preventing the onset of cognitive decline [4,12,35,42].

However, owners were described in the current study as barriers for good care because of their lack of motivation for treating the disease. Some of the free-text responses from veterinarians relating to clients as barriers include the following:

*“Clients generally deny, dismiss or decline treatment as it’s seen as ‘optional’”;*

*“I find it is the owners that don’t want to spend a lot of money on an elderly dog and don’t want to put the time in”.*

Therefore, more than being obstacles to diagnosis, owners may also interfere with the good care and implementation of treatments. This is in line with the 2023 study of Wallis et al. in which the main owner-related barrier to good care of aging dogs, perceived by veterinarians, is the owner’s awareness and willingness to act [8].

### 4.2. Attitude Towards the Disease

Another important finding is that veterinary community members are predominantly optimistic about the disease and have a good attitude towards it. Indeed, most veterinarians and veterinary nurses and technicians believe that CCD can be accurately diagnosed, that much can be done to improve the quality of life of sick patients, that the veterinary clinic has a role to play in care, and that effective treatments exist.

### 4.3. Attitude Towards Owners of Dogs with CCD

Finally, the veterinary professionals surveyed were mostly conscious of the implications of the disease, not only for the animals suffering from it but also for the owners and the caregiver burden they may undergo. Most participants felt that dementia is associated with a heavy burden of care for the owner (V: 81%, n = 59; VN/T: 77%, n = 24) and that the management of the disease should not only be focusing on the patient but also on their owner (V: 60%, n = 44; VN/T: 61%, n = 19). Indeed, the burden of care is a central feature of CCD care. Clinical levels of the burden of care in people caring for pets with serious diseases can lead to distress, anxiety, depression, and an overall decrease in the quality of life [13,43]. This is a common and frequent problem as 50% of pet owners with a serious illness [43] and 16% of caregivers of pets with CCD experience it [17]. Indeed, certain clinical signs associated with the disease can strongly affect the guardians, and owners must adapt their routine and environment to their pet, as well as administer medication. The burden of care has many implications, both on a personal level, such as emotional and psychological state and the quality of life, but also on the owner’s attitude towards others and negatively affects treatment plan adherence. It has been observed that stressed clients have more recourse to veterinary care, which increases workload, described as one of the most significant occupational stressors among veterinarians [13]. Indeed, a significant correlation has been established between caregiver burden and stress and burnout in veterinarians: a transfer of burden exists from the owners to veterinarians [44]. It is, therefore, important that members of the veterinary community are aware of the importance of caregiver burden in CCD, which is the first step to design strategies to support owners and reduce their burden.

Although most participants were aware that caregiver burden affects owners of dogs with CCD, fewer actually measure it in owners: 44% (n = 32) veterinarians and only 48% (n = 15) veterinary nurses and technicians reported that they always measure the owner’s burden of care. Owner burden assessment can be carried out by observing the owner’s attitude as certain symptoms are predictive of this syndrome [45], or more objectively by using validated scales, such as the Zarit Burden Interview (ZBI) scale, a self-assessment scale developed in human medicine that has been adapted for owners of sick animals [46]. The main limitations of care burden assessment among physicians are a lack of time and uncertainty about how to assess it [47], which surprisingly does not seem to apply to CCD as very few participants reported a lack of confidence (V: 14%, n = 10; VN/T:13%, n = 4), knowledge (V: 26%, n = 19; VN/T: 26%, n = 8), or time (V: 18%, n = 13; VN/T: 6%, n = 2) to question owners about their burden of care. The few reporting a lack of time is surprising as time limitation in consultation is often reported as an obstacle for veterinarians in managing different diseases [22,25]. However, we did not ask any questions on how they measured the burden of care, and their confidence may be misplaced. The burden of care can be reduced by accompanying the owner throughout the process, giving advice, training them in technical skills, helping to solve any problems, providing support, practicing active listening, and providing access to relevant, reliable information and external resources [48,49].

### 4.4. Limitations of the Study

The small sample size is unlikely to be representative of the entire veterinary profession in Australia and did not enable a strong statistical power. Furthermore, since the survey was limited to Australia, this limits the generalizability of the results. Given that the participation in the study was voluntary, it may have led to a representation bias. We assume that veterinarians interested in the subject or confident and familiar with the disease may be more motivated to respond to the survey and may, therefore, be more represented. Moreover, 68% (n = 21) of veterinary nurse and technician participants were involved in the management of the disease in their practice so we can assume that the population not involved in the management is underrepresented. Also, the fact that the survey was online may have participated to the selection of a younger population [50]. The two populations that emerged from our Hierarchical Cluster Analysis (Junior and Senior veterinarians) were not equally represented. Future research should aim to explore these findings further using a bigger sample size, ideally extending the study to include other countries for a broader perspective. Nevertheless, the present study provides insights into the challenges and offers a foundation for future work.

## 5. Conclusions

The present study has expanded our knowledge of the management of dogs with CCD in veterinary practice. Our main findings were that veterinarians diagnosed CCD infrequently and that few used validated scales to diagnose CCD. While a variety of treatments, such as CCD-specific medications and nutritional supplements, were used, the underdiagnosis means a large number of dogs with signs of CCD are not being effectively managed. Veterinarians that had graduated more recently were less confident in diagnosing the disease and less likely to provide treatment for CCD. Education of veterinary students and veterinary staff is needed to increase the rate of diagnosis and provide support to owners who may have a high burden of care. Working in a holistic way with veterinarians, veterinary nurses and technicians and owners will enable the quality of life of older dogs with CCD and their caregivers to be improved.

## Figures and Tables

**Table 1:** Questions relating to the management of dogs with dementia for the veterinarians and veterinary nurses/technicians. Multiple options could be selected for some questions.

Veterinarian Questions	Veterinary Nurse/Technician Questions
How do you diagnose dogs with dementia? I don’t it’s too hard I use a validated scale (e.g., DISHAA) By exclusion of other diseases (e.g., osteoarthritis) Using a specific test (e.g., cognitive test) Based on my own clinical experience Other	Are you personally involved in the management of dogs with dementia? Yes No
How often have you diagnosed CCD in a dog? A few in my career (<10) A few per year (<10 per year) A few per month (<5 per month) A few per week	No related question
Which of the following do you routinely use to treat dogs with dementia? * I don’t routinely recommend any treatment Medical specific for CCD Anti-anxiety medication Food supplements/diets Physiotherapy Referral to a dog trainer Recommend changes to their home environment Other	Which of the following does your practice use to manage dogs with dementia? * My practice does not routinely recommend any treatment Physiotherapy Referral to a dog trainer Recommend changes to their home environment I’m not sure Other
What programs does the veterinary practice/s you work in provide for older dogs? * They don’t provide specific programs for older dogs General health Obesity Other	What programs does the veterinary practice/s you work in provide for older dogs? * They don’t provide specific programs for older dogs General health Obesity Other

*: More than one option could be selected.

**Table 2:** Questions relating to attitudes to the treatment of dogs with dementia for the veterinarians and veterinary nurses/technicians. Questions that were the same for both groups are shaded in grey. Responses were a 5-item Likert scale from “strongly disagree” to “strongly agree”.

Veterinarian Questions	Veterinary Nurse/Technician Questions
I am confident in diagnosing dogs with dementia	No related question
I do not believe it is possible to accurately diagnose dementia in dogs	I do not believe it is possible to accurately diagnose dementia in dogs
I am confident in giving advice on symptom management to owners of dogs with dementia	I am confident in giving advice on symptom management to owners of dogs with dementia
I am proactive in seeking to diagnose dementia in older dogs	I think veterinarians are proactive in seeking to diagnose dementia in older dogs
I am active in treating dogs with dementia with specific medication	I think veterinarians are active in treating dogs with dementia with specific medication
I prefer treating other manageable diseases, such as arthritis and diabetes, versus dementia	I think treating other manageable diseases, such as arthritis and diabetes, versus dementia is easier for veterinarians
Much can be done to improve the quality of life of dogs with dementia	Much can be done to improve the quality of life of dogs with dementia
Managing dementia in dogs is more often frustrating than rewarding	Managing dementia in dogs is more often frustrating than rewarding
The veterinary clinic has a limited role in the care of dogs with dementia	The veterinary clinic has a limited role in the care of dogs with dementia
I don’t believe there are any effective treatments for dogs with dementia	I don’t believe there are any effective treatments for dogs with dementia
I always ask the owners of older dogs about possible signs of dementia in their dog	I always ask the owners of older dogs about possible signs of dementia in their dog

**Table 3:** Questions relating to attitudes to the owners of dogs with dementia for the veterinarians and veterinary nurses/technicians. Responses were a 5-item Likert scale from “strongly disagree” to “strongly agree”.

Questions
I always ask about the owner’s experience in looking after a dog with dementia
Dementia in dogs is associated with a large burden of care for their owners
I always measure the burden of care in caregivers of dogs with dementia
I don’t feel confident in asking owners about their feelings in caring for a dog with dementia
I don’t know how to assess caregiver burden in owners of dogs with dementia I don’t have time to ask owners of dogs with dementia about their burden of care
I think the focus should be on treating the dog with dementia and not their owner

**Table 4:** Demographics of the respondents to the survey.

	Veterinarian (n = 73)	Veterinary Nurse/Technician (n = 31)
Gender		
Female	61 (83.6%)	30 (96.8%)
Male	12 (16.4%)	1 (3.2%)
Age		
18–24	0	9 (29.0%)
25–34	33 (45.2%)	13 (41.9%)
35–44	21 (28.8%)	7 (22.6%)
45–54	12 (16.4%)	1 (3.2%)
55–64	7 (9.6%)	1 (3.2%)
Year of graduation		
2013–2023	41 (56.2%)	
2002–2012	17 (23.3%)	
1991–2001	13 (17.8%)	
1980–1990	2 (2.7%)	
Location of graduation		
Australia/New Zealand	67 (91.8%)	
Overseas	6 (8.2%)	
Type of practice		
Small animal	61 (83.6%)	
Mixed	12 (16.4%)	
Qualification postgraduation		
None	57 (78.0%)	
Membership	7 (9.6%)	
Other	5 (6.8%)	
Residency	3 (4.1%)	
Internship	1 (1.4%)	
Lived with a dog with CCD		
No	43 (58.9%)	
Yes	27 (37.0%)	
I’m not sure	3 (4.1%)	

**Table 5:** Diagnosis and management of CCD by veterinarians (n = 73).

	N (%)
How they diagnose CCD	
Own experience	27 (37.0%)
Exclusion	25 (34.2%)
Validated scale	13 (17.8%)
Specific test	3 (4.1%)
Other	2 (2.7%)
Don’t diagnose CCD	3 (4.1%)
Frequency of diagnosis	
Few in career	19 (26.0%)
Few per year	34 (46.6%)
Few per month	19 (26.0%)
Few per week	1 (1.4%)
Management of CCD	
Environmental changes	58 (79.5%)
Specific medication for CCD	57 (78.1%)
Anti-anxiety medication	53 (72.6%)
Food supplements	48 (65.8%)
Physiotherapy	12 (16.4%)
None	6 (8.2%)
Other	7 (9.6%)
Referral dog trainer	1 (1.4%)
Other treatments used (n = 34)	
Gabapentin	10 (29.4%)
Melatonin	3 (8.8%)
Hydrotherapy	2 (5.9%)
Acupuncture	2 (5.9%)
Herbal therapy	2 (5.9%)
Mirtazapine, valium, red light therapy, CBD oil	1 (2.9%)
What does the practice offer?	
General health	41 (56.2%)
None	33 (45.2%)
Obesity	22 (30.1%)
Other	5 (6.8%)

**Table 6:** Characterization of the two clusters of veterinarians (Junior group and Senior group) with v-test results for cluster-associated variables.

		Cluster	
	Overall N = 72	Junior Group N = 54 N (%), v-Test	Senior Group N = 18 N (%), v-Test
I don’t have time to ask owners of dogs with dementia about their burden of care			
Neither agree nor disagree	12 (16.6%)	12 (22.2%), 2.28	0 (0%)
Strongly disagree	15 (20.8%)	1 (1.9%)	14 (77.8%), 6.61
I don’t feel confident in asking owners about their feelings in caring for a dog with dementia			
Disagree	44 (61.1%)	40 (74.1%), 3.78	4 (22.2%)
Strongly disagree	13 (18.1%)	1 (1.9%)	12 (66.7%), 5.67
I am confident in giving advice on symptom management to owners of dogs with dementia			
Strongly agree	10 (13.9%)	1 (1.9%)	9 (50.0%), 4.56
I am proactive in seeking to diagnose dementia in older dogs			
Disagree	10 (13.9%)	10 (18.5%), 2.01	0 (0%)
Strongly agree	7 (9.7%)	0 (0%)	7 (38.9%), 4.25
I am confident in diagnosing dogs with dementia			
Strongly agree	7 (9.7%)	0 (0%)	7 (38.9%), 4.25
I don’t know how to assess caregiver burden in owners of dogs with dementia			
Neither agree nor disagree	17 (23.6%)	16 (29.6%), 2.11	1 (5.6%)
Strongly disagree	6 (8.3%)	0 (0%)	6 (33.3%), 3.85
I always ask the owners of older dogs about possible signs of dementia in their dog			
Strongly agree	2 (2.8%)	0 (0%)	2 (11.1%), 3.81
I think the focus should be on treating the dog with dementia and not their owner			
Disagree	31 (43.1%)	28 (51.9%), 2.60	3 (16.7%)
Strongly disagree	13 (18.1%)	4 (7.4%)	9 (50.0%), 3.66
The veterinary clinic has a limited role in the care of dogs with dementia			
Disagree	36 (50.0%)	33 (61.1%), 3.24	3 (16.7%)
Strongly disagree	20 (27.8%)	9 (16.7%)	11 (61.1%), 3.40
Routinely use other treatments than medication, diet, physiotherapy, referral to dog trainer, changing home environment, to treat dogs with dementia			
No	65 (90.3%)	53 (98.1%), 3.38	12 (66.7%)
Yes	7 (9.7%)	1 (1.9%)	6 (33.3%), 3.38
I always ask about the owner’s experience in looking after a dog with dementia			
Strongly agree	7 (9.7%)	1 (1.9%)	6 (33.3%), 3.38
Much can be done to improve the quality of life of dogs with dementia			
Agree	36 (50.0%)	32 (59.3%), 2.67	4 (22.2%)
Strongly agree	27 (37.5%)	14 (25.9%)	13 (72.2%), 3.37
I prefer treating dogs with other manageable diseases, such as arthritis and diabetes, versus dementia			
Agree	24 (33.3%)	23 (42.6%), 2.99	1 (5.6%)
Strongly disagree	4 (5.6%)	0 (0%)	4 (22.2%), 2.97
I am active in treating dogs with dementia with specific medication			
Strongly agree	4 (5.6%)	0 (0%)	4 (22.2%), 2.97
Routinely use physiotherapy to treat dogs with dementia			
No	60 (8.33%)	49 (90.7%), 2.63	11 (61.1%)
Yes	12 (16.7%)	5 (9.3%)	7 (38.9%), 2.63
I don’t believe there are any effective treatments for dogs with dementia			
Neither agree nor disagree	13 (18.1%)	13 (24.1%), 2.42	0 (0%)
Strongly disagree	15 (20.8%)	8 (14.8%)	7 (38.9%), 2.01
Diagnosis			
Validated scale (e.g., DISHAA, dementia symptom checker)	13 (18.1%)	6 (11.1%)	7 (39%), 2.41
Routinely use diet to treat dogs with dementia			
No	24 (33.3%)	22 (40.7%), 2.32	2 (11.1%)
Yes	48 (66.7%)	32 (59.3%)	16 (88.9%), 2.32
Dementia in dogs is associated with a large burden of care for their owners			
Strongly agree	8 (11.1%)	3 (5.6%)	5 (27.8%), 2.28
Supplemental variables			
Do you have any postgraduate specialisation?			
Membership	7 (9.7%)	2 (3.7%)	5 (27.8%), 2.69
No	57 (79.2%)	47 (87.0%), 2.61	10 (55.6%)
Age			
25–34	32 (45.8%)	28 (51.9%), 2.15	4 (22.2%)
What year did you graduate as a veterinarian?			
2013–2023	40 (55.6%)	34 (63.0%), 2.12	6 (33.3%)

## Data Availability

Data may be shared through contact with the corresponding author.
